# Microfluidic Synthesis and Purification of Magnetoliposomes for Potential Applications in the Gastrointestinal Delivery of Difficult-to-Transport Drugs

**DOI:** 10.3390/pharmaceutics14020315

**Published:** 2022-01-28

**Authors:** Carlos E. Torres, Javier Cifuentes, Saúl C. Gómez, Valentina Quezada, Kevin A. Giraldo, Paola Ruiz Puentes, Laura Rueda-Gensini, Julian A. Serna, Carolina Muñoz-Camargo, Luis H. Reyes, Johann F. Osma, Juan C. Cruz

**Affiliations:** 1 Department of Biomedical Engineering, School of Engineering, Universidad de Los Andes, Carrera 1 No. 18A-12, Bogotá 111711, Colombia; ce.torres10@uniandes.edu.co (C.E.T.); jf.cifuentes10@uniandes.edu.co (J.C.); sc.gomez11@uniandes.edu.co (S.C.G.); v.quezada@uniandes.edu.co (V.Q.); ka.giraldo@uniandes.edu.co (K.A.G.); p.ruiz@uniandes.edu.co (P.R.P.); l.ruedag@uniandes.edu.co (L.R.-G.); ja.serna10@uniandes.edu.co (J.A.S.); c.munoz2016@uniandes.edu.co (C.M.-C.); 2 Department of Chemical and Food Engineering, School of Engineering, Universidad de Los Andes, Carrera 1 No. 18A-12, Bogotá 111711, Colombia; 3 Department of Electrical and Electronic Engineering, School of Engineering, Universidad de Los Andes, Carrera 1 No. 18A-12, Bogotá 111711, Colombia

**Keywords:** magnetoliposomes, microfluidics, oral drug delivery, magnetite nanoparticles

## Abstract

Magnetite nanoparticles (MNPs) have gained significant attention in several applications for drug delivery. However, there are some issues related to cell penetration, especially in the transport of cargoes that show limited membrane passing. A widely studied strategy to overcome this problem is the encapsulation of the MNPs into liposomes to form magnetoliposomes (MLPs), which are capable of fusing with membranes to achieve high delivery rates. This study presents a low-cost microfluidic approach for the synthesis and purification of MLPs and their biocompatibility and functional testing via hemolysis, platelet aggregation, cytocompatibility, internalization, and endosomal escape assays to determine their potential application in gastrointestinal delivery. The results show MLPs with average hydrodynamic diameters ranging from 137 ± 17 nm to 787 ± 45 nm with acceptable polydispersity index (PDI) values (below 0.5). In addition, we achieved encapsulation efficiencies between 20% and 90% by varying the total flow rates (TFRs), flow rate ratios (FRRs), and MNPs concentration. Moreover, remarkable biocompatibility was attained with the obtained MLPs in terms of hemocompatibility (hemolysis below 1%), platelet aggregation (less than 10% with respect to PBS 1×), and cytocompatibility (cell viability higher than 80% in AGS and Vero cells at concentrations below 0.1 mg/mL). Additionally, promising delivery results were obtained, as evidenced by high internalization, low endosomal entrapment (AGS cells: PCC of 0.28 and covered area of 60% at 0.5 h and PCC of 0.34 and covered area of 99% at 4 h), and negligible nuclear damage and DNA condensation. These results confirm that the developed microfluidic devices allow high-throughput production of MLPs for potential encapsulation and efficient delivery of nanostructured cell-penetrating agents. Nevertheless, further in vitro analysis must be carried out to evaluate the prevalent intracellular trafficking routes as well as to gain a detailed understanding of the existing interactions between nanovehicles and cells.

## 1. Introduction

Oral drug administration is one of the most convenient routes of drug delivery due to patient preference, shelf life, sustained delivery, cost-effectiveness, and ease of large-scale manufacture [1,2]. Additionally, orally administered drugs can be directed through the gastrointestinal tract to allow localized treatment of different pathologies, such as cancer, infections, inflammations, and various digestive system diseases [1]. Nevertheless, the success of this delivery route depends on the physicochemical properties of such drugs and, particularly, their water solubility and cell-membrane permeability [3]. Different approaches have been proposed to control pharmacokinetics and improve release efficacy and safety [4]. Among these, encapsulation is one of the most attractive ones for preserving compounds with biological activity, especially when exposed to conditions that might be detrimental to their chemical stability [5,6]. In the pharmaceutical industry, the delivery of drugs has been significantly improved by encapsulation into polymeric capsules and liposomes [7,8]. These liposomal vehicles have been widely studied for pharmaceutical preparations with limited passing across biological barriers, such as the blood–brain barrier and the intestinal epithelium, due to attractive features such as the flexibility of changing their chemical composition, structure, and colloidal size [9,10,11,12,13].

Additionally, a significant challenge in drug delivery has been to achieve better internalization and high bioavailability [9,10]. This challenge has been addressed by an increasing number of delivery vehicles that include both viral and non-viral vectors [11]. Among these, magnetite nanoparticles (MNPs) functionalized with translocating proteins and peptides have been studied as potent vehicles for cell penetration and endosomal escape. Moreover, a possible enhancement of escape is expected if these vehicles are encapsulated into liposomes [12,14,15,16].

Liposomes with encapsulated MNPs, called magnetoliposomes (MLPs), have been extensively used as carriers in the pharmaceutical industry due to their ability to release various active molecules at a given site without the need for molecularly targeted agents [17]. This is in addition to the improvement in the biocompability, drug delivery rate for some compounds, and cellular uptake without a significant reduction in the activity of the functional compounds immobilized and delivered employing MNPs [18,19]. Moreover, these novel drug delivery vehicles might offer potential improvements in targeting, stabilization of antimicrobial agents, and gastroretention. This might help to reduce various possible side effects of oral administration, such as the uncontrolled destruction of both pathogenic and non-pathogenic microbiota, and, therefore, prevent the appearance of complications such as dysbiosis [20]. This correlation between the microbiota’s metabolic activity and the improvement of the bioavailability is particularly relevant in determining the overall efficacy of these novel drug delivery vehicles. Therefore, it is important to determine if the carrier nanovehicle can increase the compound bioavailability and reduce the associated biotransformations, which in turn define the bioactivity expression in response to a particular microbiome [21,22,23].

Currently, MLPs have been explored as drug delivery carriers to treat conditions as diverse as cancer, Parkinson’s, and Alzheimer’s [24,25,26]. Over the past few years, several techniques have been proposed for preparing MLPs, in which nanoparticles can be encapsulated in the aqueous lumen, embedded in the lipid bilayer, or conjugated on the surface of the liposome [27,28,29,30]. By implementing these techniques, liposome solutions’ parameters vary considerably, posing some challenges related to their particle size, dispersity, lamellarity, entrapment efficiency, and, most importantly, the difficulty in separating the non-encapsulated/unbound MNPs [31,32].

Currently, liposome preparation techniques using microfluidic systems have allowed greater control over physical properties, yielding massive and robust production of MLPs with uniform size distribution, high loading efficiencies, and reduced costs [33,34,35]. Nevertheless, there is still a challenge in the separation and sample purification methods due to the minor size differences between the MLPs and the non-encapsulated nanoparticles [32,36]. Due to several limitations of current separation techniques, microfluidic systems have been proposed as potential low-cost particle separation systems based on different active or passive methods to separate nanoscale objects such as DNA, viruses, proteins, exosomes, and nanoparticles [37]. Exploring the scope of microfluidics separation approaches, there has been a growing interest in using magnetic gradients to retain excess MNPs without compromising the integrity of the MLPs. The significant traction gained by this approach could be mainly attributed to its applicability, versatility, and ease of implementation in many areas of the biomedical field, including disease diagnostics, therapeutics, and cell sorting [38,39,40].

This study proposes the synthesis of MLPs using a microfluidic approach; FEM simulations implemented in COMSOL Multiphysics^®^ to study the separation of MLPs from nanoconjugates aided by a magnetophoretic microfluidic system; the manufacture and experimental validation of two separation devices; and, finally, the in vitro testing of the synthesized MLPs to evaluate whether this delivery vehicle is biocompatible and improves the cell penetration of orally administered CefTRIAxone, a drug with exceedingly low intestinal absorption. The prototypes of the microfluidic devices for MLPs synthesis were manufactured by a low-cost method based on laser cutting techniques and led to MLPs with acceptable physical properties and encapsulation efficiency. Additionally, the separation devices showed different efficiencies depending on the implemented approach which varied considerably compared with those obtained during the experimental validation. Nevertheless, qualitatively, both methods led to similar results, confirming their suitability for the intended objective. Finally, the preliminary in vitro evaluation demonstrated that MLPs showed high biocompatibility, low endosomal entrapment, and high internalization rates, which are crucial factors in developing novel vehicles for delivering difficult-to-transport drugs.

## 2. Materials and Methods

### 2.1. Magnetite Nanoparticles Synthesis and Functionalization

**Scheme 1:**
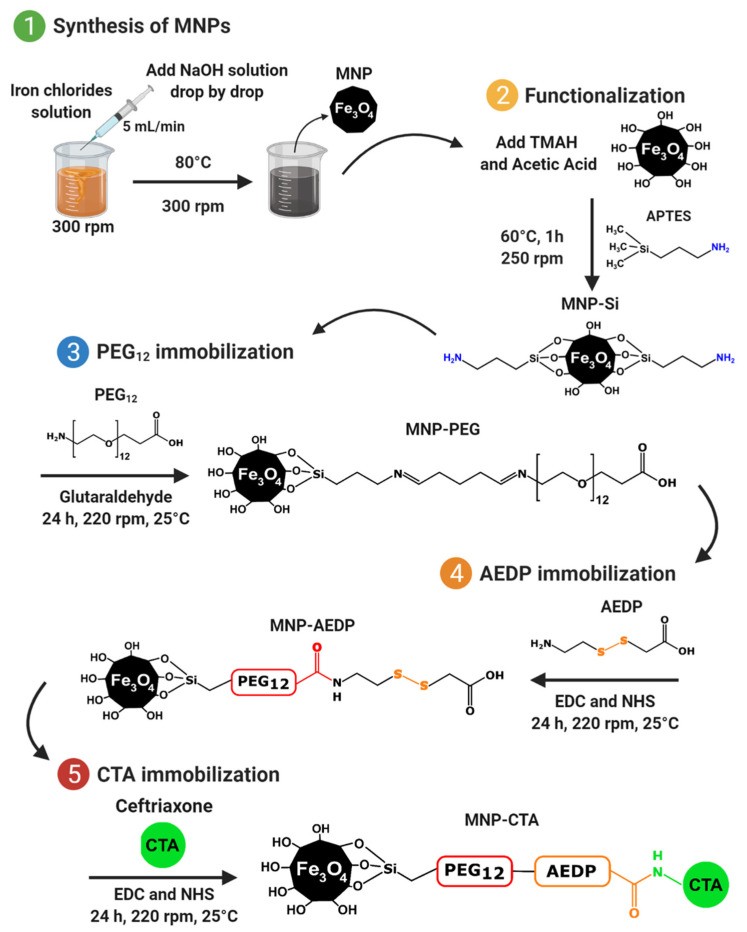
Schematic of the developed workflow for the synthesis of MNP-PEG-AEDP-CTA nanoconjugate. (1) Magnetite nanoparticles (MNPs) are synthesized by co-precipitation. (2) MNPs are silanized with APTES and subsequently with (3) NH_2_-PEG-propionic acid. This was followed by the conjugation of (4) AEDP and, finally, (5) the immobilization of the drug CTA.

Magnetite nanoparticles (MNPs) were synthesized by the chemical co-precipitation method. For this, FeCl_2_ (0.34 g, J. T. Baker, Phillipsburg, NJ, USA) and FeCl_3_ (0.93 g, Merck, Kenilworth, NJ, USA) were solubilized in 60 mL of type I water. In addition, 0.69 g of NaOH (PanReac AppliChem, Darmstadt, Germany) was added to 17 mL of type I water, and then both solutions were heated at 80 °C. Next, a NaOH solution was added dropwise to the iron chloride solution at a 5 mL/min rate under constant stirring. A black precipitate was observed, corresponding to the formation of MNPs. The obtained MNPs were then washed four times with NaCl solution (1.5% *w*/*v*) and twice with type I water aided by a neodymium magnet. Then, 100 mg of MNPs were silanized by adding 50 µL of glacial acetic acid (PanReac AppliChem, Barcelona, Spain) followed by 400 µL of (3-aminopropyl) triethoxysilane (APTES, 98%, Sigma-Aldrich, St. Louis, MO, USA). The MNPs solution was left to react under constant stirring (250 rpm) at 60 °C for 1 h and then washed as mentioned previously. Later, 100 mg of the silanized MNPs (MNP-APTES) was mixed with 2 mL of glutaraldehyde solution (2% *v*/*v*, Sigma-Aldrich, St. Louis, MO, USA) (solution previously stirred (220 rpm) at room temperature for 1 h to allow glutaraldehyde activation (MNP-APTES-GA)). Then, 5 mL of a NH_2_-PEG-propionic acid (99%, Merck, Darmstadt, Germany) solution (2 mg/mL) was added dropwise to the MNP-APTES-GA conjugates under constant stirring to obtain MNP-APTES-PEG conjugates. The solution was left to react at 220 rpm and room temperature for 24 h and washed four times with NaCl solution (1.5% *w*/*v*) and twice with type I water. Similarly, (3-[(2-aminoethyl)dithio]propionic acid) (AEDP, ThermoFisher, Waltham, MA, USA) immobilization was carried out using 14 mg of *N*-[3-dimethylammino)-propyl]-*N*′-ethyl carbodiimide hydrochloride (EDC, 98%, Sigma-Aldrich, St. Louis, MO, USA) and 7 mg of *N*-hydroxy succinimide (NHS, 98%, Sigma-Aldrich, St. Louis, MO, USA) solution in 5 mL of type I water added to 100 mg of MNP-PEG in 50 mL of type I water to activate the terminal carboxyl groups. Nanoparticles were ultrasonicated (ultrasonic bath, Branson, Danbury, CT, USA) for 10 min, and 5 mL of an AEDP solution (5 mg/mL) was added dropwise under constant stirring. The solution was left to react at 220 rpm and room temperature for 24 h. The MNP-PEG-AEDP conjugates were washed with NaCl (1.5% *w*/*v*) and type I water. Finally, the antibiotic CefTRIAxone (CTA, Vitalis, 1 g I.M/I.V) was immobilized by following the same protocol for AEDP immobilization, using 5 mL of CefTRIAxone solution (2 mg/mL). Scheme 1 shows the complete methodology for the synthesis.

**Figure 1:**
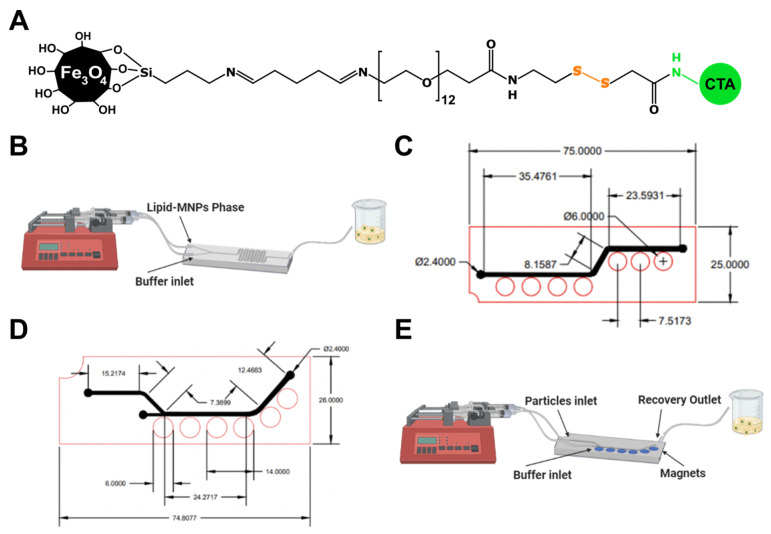
(**A**) Schematic of the synthesized nanoconjugate for the MLPs production. (**B**) Microfluidic experimental setup for the synthesis phase. Microfluidic separation designs proposed: (**C**) System 1 and (**D**) System 2. (**E**) Microfluidic experimental setup for the separation phase.

The resulting MNP-PEG-AEDP-CTA conjugates were labeled with rhodamine B (95%, Sigma-Aldrich, St. Louis, MO, USA) for fluorescence-based assays. For this, 14 mg of EDC, 7 mg of NHS, and 5 mg of rhodamine B (RdB) were dissolved in 5 mL of type I water containing 2 mL of dimethylformamide (DMF, Supelco/Sigma-Aldrich, Bellefonte, PA, USA). Rhodamine B solution was left under constant stirring for 15 min to allow the activation of carboxylic groups. Next, the previously activated rhodamine B solution was added to 50 mL of MNP-PEG-AEDP-CTA aqueous solution (2 mg/mL) and left to react at 220 rpm, room temperature, and in complete darkness for 24 h. The resulting MNP-PEG-AEDP-CTA-RdB was washed several times with NaCl (1.5% *w*/*v*) and type I water to remove the excess reagents. MNP-PEG-AEDP-CTA-RdB nanoconjugates were resuspended in type I water and stored in complete darkness at 4 °C until further use for the MLPs preparation described below. A schematic of the synthesized nanoconjugate is shown in Figure 1A.

### 2.2. Magnetoliposomes Synthesis Using the Microfluidic Approach

#### 2.2.1. Lipidic-MNPs Phase Preparation

First, 100 mg of soy lecithin (1-α-lecithin, soybean-cas 8002-43-5-calbiochem) (Merck, Kenilworth, NJ, USA) was dissolved in 10 mL of chloroform c2432 (>99.5%, Merck, Kenilworth, NJ, USA), and 1 mL and 2 mL of MNPs (1.7 mg/mL) were added. The sample was rotary evaporated for 1 h at 45 °C under a vacuum (rotary evaporator, Hei-VAP Value Digital Vertical, Heidolph, Schwabach, Germany). Then, 10 mL of ethanol (96% *v*/*v*) was added to the rotary evaporator flask. The sample was vigorously agitated and rotated for 30 min at atmospheric temperature and pressure.

#### 2.2.2. Microfluidic System Manufacture and Experimental Setup

The manufacture and design of microfluidic devices for MLP synthesis was based on the study presented by Aranguren et al. [41]. A laser cutting machine (TROTEC ^®^ Speedy 100, 60 w laser cutter, TROTEC, Marchtrenk, Austria) was used for engraving and cutting the microfluidic channels proposed on a PMMA substrate. The two or three layers of the microfluidic devices were manually aligned and sealed using 96% (*v*/*v*) ethanol with a mechanical press placed on a hot plate (110 °C). For the synthesis, the microfluidic channels were purged with a 10 mL syringe filled with 96% (*v*/*v*) ethanol for 15 min. Then, a syringe filled with the lipidic-nanoconjugates phase and a syringe with NaCl (anhydrous, Redi-Dri™, free-flowing, ACS reagent, >99%) solution (0.05 M) were mounted on an infusion pump (MedCaptain MP30), as is shown in Figure 1B. The syringes were connected to the microsystems using two probes (Nelaton, Probes, Medex caliber 8) (Medex, Smiths Medical Inc., Minneapolis, MN, USA). The synthesis was carried out using a total flow ratio (TFR) set at 2.5 mL/min and 5 mL/min with a varying flow rate ratio (FRR) from 1:1 to 5:1 (aqueous:solvent ratio).

### 2.3. Magnetoliposomes Characterization

Magnetoliposome size and polydispersity index were measured using the Zetasizer Nano ZS (Malvern, Panalytical, Egham, UK). Additionally, a morphology and size characterization using a TEM Tecnai F20 Super Twin TMP (FEI, Hillsboro, OR, USA) was performed to determine the synthesis effectiveness. For the TEM analysis, a sample drop was deposited on a copper grid with a carbon coating that was dried for 1 h. Next, the prepared sample was stained with 2% uranyl acetate by depositing one drop on the grid for 8 min, washed with deionized water, and left to dry for imaging at a total magnification of 71, 97, and 145 kX.

### 2.4. Magnetolipsomes Encapsulation Efficiency (EE%)

The encapsulation efficiency of synthesized MLPs was analyzed using a Spectrofluorometer (0239D-2219 FluoroMax plus C, Horiba, Miyanohigashi, Japan) to track changes in the fluorescence intensity before and after treatment with Triton X-100 (Sigma-Aldrich, St. Louis, MO, USA). For this characterization, 100 µL of MLPs prepared with MNP-PEG-AEDP-CTA-RdB was pipetted into a 96-well microplate for the first fluorescence-based analysis where the fluorescence intensity was measured. Then, 10 mL of Triton X-100 was added to the microplates to break the magnetoliposome membranes and allow the labeled nanoconjugates to escape. Finally, a second measure of the sample fluorescence intensity was carried out to analyze the changes compared with the initial intensity. The fluorescence spectrum of rhodamine B allowed tracking intensity by setting up excitation and emission filters at 546 nm and 568 nm, respectively. The encapsulation efficiency was calculated using Equation (1):

(1) $$\mathrm{EE}\;(\%)=100\;\times \;\frac{\left(\mathrm{Int}\;(\mathrm{Final})\;-\;\mathrm{Int}\;(\mathrm{Initial})\;-\;\mathrm{Int}\;(\mathrm{Triton}\;\text{X-100})\right)}{\mathrm{Int}\;(\mathrm{Final})},$$

where *Int (Final)* is the emission post-Triton *X-100, Int (Initial)* is the emission pre—*Triton X-100* treatment, and *Int (Triton X-100)* is the blank emission of *Triton X-100*.

### 2.5. Magnetoliposomes Purification

#### 2.5.1. Lipidic-Nanoconjugates Phase Preparation

The design of the two components of the microfluidic devices proposed (System 1 and System 2) was conducted on two rectangular PMMA layers (width: 7.48 cm, height: 2.60 cm) via AutoCAD v23.0 (AutoDesk Inc., San Rafael, CA, USA). The microfluidic channel was superimposed over one of the two layers, followed by locating holes in each piece. The larger holes were to accommodate permanent neodymium magnets 6 mm in diameter and 8 mm deep, while the smaller ones were for the inlets and outlets of the microchannel. In this case, the diameter was 2.4 mm. (Figure 1C,D).

In these designs, the magnetophoretic separation was analyzed in silico with two different methods. The first one involved implementing the particle tracing module, where magnetoliposomes and nanoconjugates were considered separate components within the microfluidic device. The simulation was conducted by implementing the laminar flow, particle tracing, and the magnetic field without current modules of COMSOL Multiphysics^®^ (COMSOL Inc., Stockholm, Sweden). The second method was based on a mixture model approach to simulate a dispersed phase, considered a ferrofluid (i.e., a suspension of magnetic nanoconjugates in water) due to the high concentration of nanoparticles in the domain. Several recent reports support this assumption (rather than considering individual particles) for FEM simulations, since it leads to results that are closer to those obtained experimentally [42,43,44,45,46]. In this case, the implemented COMSOL modules were the “mixture model with laminar flow”, the “magnetic field without current”, and “diluted species’ transport”.

#### 2.5.2. Multiphysics Simulations of Magnetophoretic Separation via the Particle Tracing Module

The magnetic field was incorporated into the simulations through the “magnetic fields with no currents” model of the AC/DC module of COMSOL. In this case, the magnetic field intensity is calculated by solving the Maxwell equations for permanent magnets, resulting in the governing equations shown in Equations (2)–(4) [47].

H = −∇ V_m_, (2)

∇ · B = 0,(3)

where:

(4) $$B=\mu_{0}\mu_{r}\vec{H\;}+B_{r},$$

where $\vec{H\;}$ is the magnetic field distribution, V_m_ is the magnetic scalar potential, B is the magnetic flux density distribution, and μ_0_ and μ_r_ the vacuum permeability and relative permeability. Finally, B_r_ represents the remanent flux density that, in this case, was set to 1 T. The second physic coupled to the model was the laminar flow governed by the Navier–Stokes conservation of momentum equation for incompressible fluids, Equation (5), which is accompanied by the conservation of mass by the continuity equation (Equation (6)).

∇[−PI + μ (∇u + (∇u)^T^)] + F = 0, (5)

ρ∇ · (u) = 0,(6)

where P is the pressure, µ is the fluid’s dynamic viscosity, F accounts for the volumetric forces, and ρ is the fluid density. Finally, the particle tracing for fluid flow was coupled to the model, where Newton’s second law governs the movement of the particles transported within the device. This is described by Equation (7):

(7) $$\frac{{d(m}_{p}\upsilon )}{\mathrm{dt}}=F_{t},$$

where m_p_ is the mass of the particles, υ the velocity, and F_t_ the sum of all forces acting on the particle. In this case, the involved forces were the drag force, defined by Equations (8) and (9) by the Stokes law, and the magnetic force, dependent on the magnetic flux density distribution described by Equation (10).

(8) $$F_{D}=\frac{1}{\tau_{p}}m_{p}(u-\upsilon ),$$

(9) $$\tau_{p\;}=\frac{\rho_{p\;}d_{p}^{2}}{18\mu },$$

where m_p_ is the particle mass, u is the velocity field, υ the particle velocity, ρ_p_ the particle density, d_p_ the particle diameter, and μ the dynamic viscosity.

(10) $$F_{M\;}=\frac{V_{m}\Delta X}{\mu_{0}}(B\cdot \nabla )B$$

where ∆X is the magnetic susceptibility difference between the particle and the fluid. Finally, the FEM simulations to solve the set of equations for laminar flow and magnetic field were conducted via a stationary study. Additionally, for the particle tracing module for 1200 particles per component (i.e., nanoconjugates and MLPs), a bi-directionally coupled particle tracing was used with a MUMPS solver. The computational domain was meshed with 176,141 domain elements and 6523 boundary elements for System 1 and 47,152 domain elements and 1838 boundary elements for System 2. This module’s boundary conditions were the drag force in all the microfluidic channel domains and the system’s inlets as the main entrance for the particles into the system. The model parameters are summarized in Table 1:

#### 2.5.3. Multiphysics Simulations of Magnetophoretic Separation via the Mixture Model

The magnetic field was established using a magnetic field, no currents physic, as described previously. The governing equations for this simulation are presented in Equations (1)–(3). The ferrofluid was simulated, aided by the mixture model, laminar flow physics. The interface solves a set of Navier–Stokes equations for the momentum of the mixture. The pressure distribution is calculated from a mixture-averaged continuity equation, and the velocity of the dispersed phase is described by a slip model [48]. The momentum conservation equation and the continuity equation are presented in Equations (11) and (12):

(11) $$\;\rho \frac{\mathrm{du}}{\mathrm{dt}}+\rho (u\;\cdot \;\nabla )u=[-\text{pl}+\mu (\nabla u+{\left(\nabla u\right)}^{T}-\frac{2}{3}(\nabla \;\cdot \;u)]-\nabla \cdot \;[\rho C_{d}(1-C_{d})U_{\mathrm{slip}}{U_{\mathrm{slip}}}^{T}]+F,$$

(12) $$(\rho_{c}-\rho_{d})\;\{\nabla \;\cdot \;[\Phi_{d}\;(1-C_{d})\;U_{\mathrm{slip}}]+\frac{m_{\mathrm{dc}}}{\rho_{d}}\}+\rho_{c}\;(\nabla \;\cdot \;u)=0,$$

where P is the pressure, μ is the dynamic viscosity of the fluid, ρ_c_ and ρ_d_ the continuous phase density and dispersed phase density, Φ_d_ the volume fraction of the dispersed phase, m_dc_ the turbulent dispersed phase diffusion, and F the body forces, which in this case are described by the Kelvin body force due to a spatially non-uniform magnetic field according to Equation (13) [46]:

(13) $$F=(\vec{M}\cdot \nabla )\vec{B},$$

where $\vec{B\;}$ is the magnetic flux density distribution and $\vec{M\;}$ the magnetization. Finally, the diluted species’ transport was used to determine the effective concentration of the nanoparticles inside the channel. This solution inside the microchannel is described by the convective–diffusive Equation (14).

(14) $$\frac{{\mathrm{dC}}_{p}}{\mathrm{dt}}+\nabla \cdot (-D_{p}C_{p})+u\cdot \nabla {\;C}_{p}=0,$$

where C_p_ is the concentration, u is the velocity field provided by the mixture model, and D_p_ is the effective diffusivity of the NPs as calculated by Equation (15).

(15) $$D_{p\;}=\frac{K_{B}T_{\;}}{{3\Pi \eta }_{\mathrm{ff}}d_{p}},$$

Here, K_B_ is the Boltzmann constant, T is temperature, η_ff_ is the ferrofluid viscosity, and d_p_ is the diameter of the particles.

Finally, time-dependent simulations were carried out using a MUMPS solver with a phase volume fraction of 0.2 for each particle component entering the upper inlet. For System 1, complete mesh consists of 59,862 domain elements and 1845 boundary elements, while for System 2, the computational domain mesh comprised 66,735 domain elements and 1946 boundary elements. The final meshing is shown in Supplementary Figure S2, and the parameters used for this model are presented in Table 2.

#### 2.5.4. Microfluidic System Manufacture and Experimental Setup

The manufacture of the microfluidic device for the MLPs synthesis (Supplementary Figure S1) was based on the methodology presented above and reported previously by us in the studies of Aranguren et al. and Campaña et al. [41,49]. For the first design, seven neodymium magnets were in proximity to the microchannels, as is shown in Figure 1C. In parallel, one syringe of 10 mL was filled with the solution of LPs and MNP-PEG-AEDP-CTA nanoconjugates and connected to the system inlet. The solution was pumped into the device with syringe pumps (78-8110C Programmable Touch Screen, Cole-Parmer^®^, Vernon Hills, IL, USA, and B Braun Perfusor^®^ compact, B. Braun, Melsungen, Germany) at total flow rates (TFRs) from 1 to 3 mL/min. The second design included six neodymium magnets, as is shown in Figure 1D. Two syringes of 10 mL were filled up and connected to the system’s inlets. The first one was filled with the LPs and MNP-PEG-AEDP-CTA nanoconjugates, while the second one with a NaCl solution (0.05 M) (Figure 1E). The solutions were pumped into the device with the syringe pumps at total flow rates from 1 to 3 mL/min by maintaining a 1:1 FRR. The samples recovered from each design were analyzed using a spectrofluorometer (0239D-2219 FluoroMax plus C, Horiba, Miyanohigashi, Japan) to track changes in the fluorescence intensity compared with the control sample, which is the solution before injection into the system. As for the EE experiment, the fluorescence spectrum of rhodamine B allowed intensity tracking by setting up excitation and emission filters at 546 nm and 568 nm, respectively.

### 2.6. In Vitro Testing of MLPs

#### 2.6.1. Hemocompatibility

To determine the hemocompatibility of the liposomes, MNP-PEG-AEDP-CTA nanoconjugates, and magnetoliposomes, a blood sample was extracted from a healthy human donor in a vacutainer tube containing EDTA. Erythrocytes were obtained by centrifugation at 1800 rpm for 5 min. The supernatant was discarded, and erythrocytes were washed five times with NaCl solution (0.9% *w*/*v*) and twice with PBS 1×. To form a stock solution, 1 mL of the washed erythrocytes was suspended in 9 mL of PBS 1× and carefully homogenized. The liposomes and magnetoliposomes were evaluated at 0.1, 0.05, and 0.025 mg/mL, while MNP-PEG-AEDP-CTA nanoconjugates were at concentrations ranging from 200 µg/mL to 12.5 µg/mL. Triton 100-X (10% *v*/*v*) and PBS 1× were used as positive and negative controls, respectively. To evaluate the hemolytic activity, 100 µL of the erythrocyte stock solution was seeded with 100 µL of the different treatments in a 96-well microplate. The microplate was then incubated under constant stirring at 37 °C for 1 h. The plate was centrifuged, and the supernatants were then transferred to another 96-well microplate. Finally, absorbance was read at 450 nm, and hemolysis percentage was calculated by following Equation (16):

(16) $$\mathrm{Hemolysis}\;(\%)=100\;\times \;\frac{\left(\mathrm{Abs}\;(\mathrm{sample})\;-\;\mathrm{Abs}\;(C-)\right)}{\left(\mathrm{Abs}\;(C+)\;-\;\mathrm{Abs}\;(C-)\right)}$$

#### 2.6.2. Platelet Aggregation

The platelet aggregation capacities of the liposomes, MNP-PEG-AEDP-CTA, and magnetoliposomes were evaluated by exposing them to a blood sample extracted from a healthy human donor in a vacutainer tube containing sodium citrate. Platelet-rich plasma (PRP) was obtained by centrifuging the collected blood at 1000 rpm for 15 min. Erythrocytes were discarded, and the supernatant containing PRP was used to run the test. The liposomes and magnetoliposomes were evaluated at 0.1, 0.05, and 0.025 mg/mL and the MNP-PEG-AEDP-CTA at concentrations ranging from 200 µg/mL to 12.5 µg/mL. Thrombin and PBS 1× were used as positive and negative references, respectively. The aggregation capacity was evaluated by exposing 50 µL of PRP to 50 µL of the different treatments in a 96-well microplate. The microplate was incubated at 37 °C for 5 min, and then absorbance was read at 620 nm. Platelet aggregation percentage was calculated by following Equation (17):

(17) $$\mathrm{Platelet}\;\mathrm{aggregation}\;(\%)=100\;\times \;\frac{\mathrm{Abs}\;(\mathrm{sample})}{\mathrm{Abs}\;(C+)},$$

#### 2.6.3. Cytotoxicity

The cytocompatibility of the liposomes, MNP-PEG-AEDP-CTA, and magnetoliposomes was determined as a measure of the impact on the metabolic activity in two different cell lines, namely, Vero (ATCC^®^ CCL-81) and gastric cancer (AGS, ATCC^®^ CRL-1739) cells, with the aid of a colorimetric assay based on 3-(4,5-dimethylthiazol-2-yl)-2,5-diphenyltetrazolium (MTT, Sigma-Aldrich, St. Louis, MO, USA). The liposomes and magnetoliposomes were evaluated at 0.1, 0.05, and 0.025 mg/mL and the MNP-PEG-AEDP-CTA at serial dilutions from 200 µg/mL to 12.5 µg/mL. Non-supplemented DMEM medium was used as the negative control. To evaluate cell viability in the different cell lines, 100 µL of a cell stock solution in DMEM medium supplemented with FBS (10%) was seeded in a 96-well microplate at a cell density of 10 × 10^4^ cells/well. Microplates were incubated at 37 °C, 5% CO_2_, and a humidified atmosphere for 24 h. After that, DMEM medium supplemented with FBS (10%) was extracted and replaced with a non-supplemented DMEM medium containing the different treatments. Viability was studied at 24 and 48 h after the exposure. To determine the viability percentage, 10 µL of MTT reagent (5 mg/mL) was added to each well, and the microplates were then incubated, under the same conditions described above, for 2 h. Finally, supernatants were discarded, and 100 µL of DMSO was added to each well to dissolve the formed formazan crystals. The absorbance was read at 595 nm with the aid of a microplate reader (Thermo Scientific Multiskan™ FC Microplate Photometer). Cell viability was calculated by following Equation (18):

(18) $$\mathrm{Cell}\;\mathrm{viability}\;(\%)=100\;\times \;\frac{\mathrm{Abs}\;(\mathrm{sample})}{\mathrm{Abs}\;(C-)},$$

#### 2.6.4. Cell Internalization and Endosomal Escape Analysis

Cell internalization and endosomal escape abilities of MNP-PEG-AEDP-CTA-RdB nanoconjugates and magnetoliposomes were assessed by colocalization between the labeled nanoconjugates and Lysotracker Green^®^ DND-26 (Thermo Fisher, Waltham, MA, USA) in Vero (ATCC^®^ CCL-81) and gastric cancer cells (AGS, ATCC^®^ CRL-1739). For this, cells were seeded on glass slides deposited into a 24-well microplate at a cell density of 5 × 10^4^ cells/well. Cells were then incubated with DMEM medium supplemented with FBS (10% *v*/*v*) at 37 °C and 5% CO_2_ for 24 h to allow cell adhesion. Once the incubation time was achieved, DMEM medium was extracted and replaced with supplemented DMEM medium containing the different treatments at 50 µg/mL and magnetoliposomes with an equivalent amount of MNP-PEG-AEDP-CTA-RdB of 25 µg/mL. Cells were incubated for 0.5 h and 4 h. Next, the medium was extracted, and cells were washed three times with PBS 1× to remove the excess of the treatments. After this, PBS 1× was drawn, and cells were exposed to a DMEM solution containing Hoechst 33,342 (Thermo Fisher, Waltham, MA, USA) (1:1000) and Lysotracker Green^®^ DND-26 (1:10000) for 10 min before imaging via confocal microscopy. The images were acquired in an Olympus FV1000 confocal laser scanning microscope with a PlanApo 60× oil immersion objective. Imaging of nuclei, endosomes, and MNP-PEG-AEDP-CTA-RdB nanoconjugates was performed at the following excitation/emission wavelengths: 358 nm/461 nm, 488 nm/520 nm, and 546 nm/575 nm, respectively. Analysis was carried out by taking 10 images for each treatment with an average of 10 cells per image. The internalization and cytosol distribution were studied by calculating the surface area coverage. Image processing and analyses were performed on the software Fiji-ImageJ^®^. Statistical analyses and data processing were carried out on GraphPad Prism^®^ V 6.01 software (GraphPad Software, La Jolla, CA, USA). Statistical comparisons were made using the unpaired t-test. Results of *p* ≤ 0.05 (*) were considered significant.

### 2.7. Statistical Analyses

All data measurements are reported as mean ± standard deviation. Each experiment was carried out in triplicate. Data analysis was performed using the Graph Pad Prism V 6.01^®^ software. Statistical comparisons were determined by running two-way ANOVA followed by post-treatment (Dunn’s Multiple Comparison test). Results with *p*-value ≤ 0.05 (*) were considered significant. (*) corresponds to statistically significant difference with a *p*-value between 0.01 and 0.05; (**) represents 0.001 ≤ *p*-value < 0.01; (***) represents 0.0001 ≤ *p*-value ≤ 0.001; and (****) represents *p*-value < 0.0001. In addition, “ns” represents no statistically significant differences between the treatments.

## 3. Results and Discussion

### 3.1. Characterization of Magnetoliposomes Using the Microfluidic Approach

**Figure 2:**
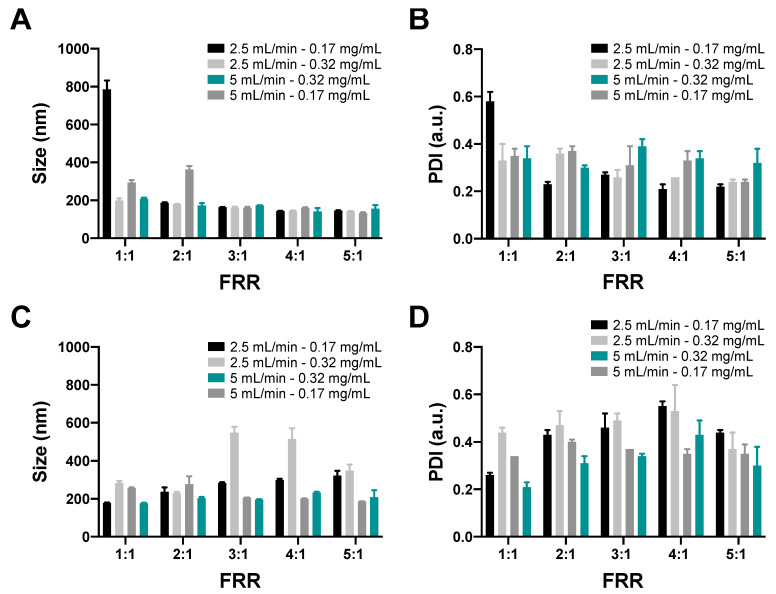
MLPs size and PDI for the two-layer device and the three-layer device. Two-layer device: (**A**) MLPs size using TFRs of 2.5 mL/min and 5 mL/min for nanoconjugates concentrations of 0.17 mg/mL and 0.32 mg/mL and FRRs from 1:1 to 5:1. (**B**) MLPs PDI using TFRs of 2.5 mL/min and 5 mL/min for nanoconjugates concentrations of 0.17 mg/mL and 0.32 mg/mL and FRRs from 1:1 to 5:1. Three-layer device: (**C**) MLPs size using TFRs of 2.5 mL/min and 5 mL/min for nanoconjugates concentrations of 0.17 mg/mL and 0.32 mg/mL and FRRs from 1:1 to 5:1. (**D**) MLPs PDI using TFRs of 2.5 mL/min and 5 mL/min for nanoconjugates concentrations of 0.17 mg/mL and 0.32 mg/mL and FRRs from 1:1 to 5:1.

Figure 2A,B shows the size and PDI of the synthesized MLPs using the two-layer device. In this case, no apparent differences in size were identified for the evaluated TFRs and the concentration of the nanoconjugates used in the experiment for different FRRs. Nevertheless, there is a slight decrease in size with the increase of the FRR in almost all cases except for the TFR of 5 mL/min at a concentration of nanoconjugates of 0.17 mg/mL for the 1:1 to the 2:1 FRR, where it is comparable with the results for the synthesis of liposomes for different FRRs [31,41]. Additionally, the size of the MLPs synthesized is smaller than 400 nm except for the case where the TFR, concentration, and FRR are the lowest. The PDI of the synthesized MLPs presented values under 0.5 in almost all cases, which indicates that the MLPs samples obtained had acceptable polydispersity.

Figure 2C,D shows the size and PDI of the synthesized MLPs using the three-layer device. In this case, there is a significant difference in size for the evaluated TFRs and the concentration of the nanoconjugates used in the experiment for different FRRs. The 5 mL/min TFR led to lower size values than the ones obtained using the 2.5 mL/min TFR, and there is a slight increase in the size for the MLPs at a higher concentration of nanoconjugates. These observations are contrary to those reported by Joshi et al., according to which the TFR has no impact on the liposome sizes [33]. The dimensions of the channels and the incorporation of nanoconjugates into the lipid phase before entering the system might be relevant factors to explain the identified differences. The obtained MLPs sizes are larger than those obtained using the two-layer device, but in this case such sizes are under 400 nm. Additionally, the PDI values of the MLPs were below 0.5 in nearly all cases, which indicates that the MLPs samples obtained have an acceptable polydispersity. Compared with the two-layer device, there is a slight increase in the PDI values. This strongly suggests the two-layer system provides superior control over MLP sizes and their PDI values.

**Figure 3:**
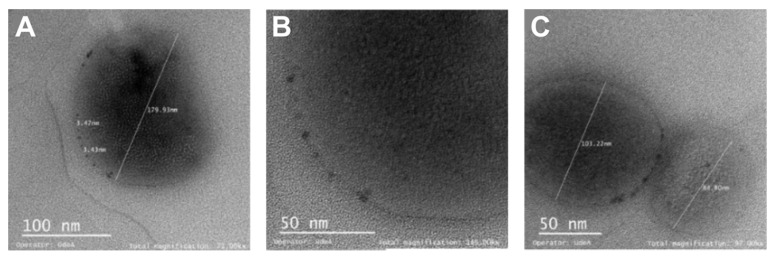
MLP characterization via TEM. (**A**) MLP synthesized with the three-layer device using TFR of 5 mL/min for nanoconjugates concentration of 0.32 mg/mL and a FRR set at 4:1 (**B**) Magnification by 145 k× of the MLP presented in A. (**C**) MLPs synthesized with the two-layer device using TFR of 5 mL/min for nanoconjugates concentration of 0.32 mg/mL and a FRR set at 4:1.

Figure 3 shows the TEM characterization of the MLPs obtained with both the two-layer and three-layer devices. The images show that the MLPs formed correctly and in agreement with previous reports of MLPs synthesized via microfluidics [34,35]. The images also show that the size of the MLPs obtained for the three-layer device is slightly larger than that obtained with the two-layer device, which agrees well with the hydrodynamic diameters measured via DLS.

### 3.2. Magnetolipsomes Encapsulation Efficiency

**Figure 4:**
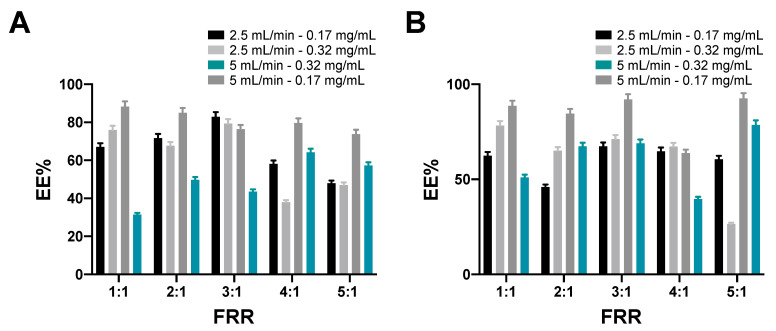
MLP encapsulation efficiency (EE%) for two-layer and three-layer devices. (**A**) Encapsulation efficiency for the MLPs synthesized using the two-layer device with TFRs of 2.5 mL/min and 5 mL/min at nanoconjugates concentrations of 0.17 mg/mL and 0.32 mg/mL and FRRs from 1:1 to 5:1. (**B**) Encapsulation efficiency for the MLPs synthesized using the three-layer device with TFRs of 2.5 mL/min and 5 mL/min at nanoconjugates concentrations of 0.17 mg/mL and 0.32 mg/mL and FRRs from 1:1 to 5:1.

Figure 4 shows the encapsulation efficiencies (EE%) for the MLPs synthesized with both devices at different TFR values, nanoconjugates concentrations in the lipid phase, and FRRs. The EE% obtained at 0.17 mg/mL nanoconjugates concentration with the two-layer device was higher for almost all evaluated FRRs, while no identifiable trend was observable for the three-layer device. This agrees well with the notion that a superior control of MLP assembly is achievable with the two-layer devices. Nevertheless, the results show efficiencies ranging from 20% to 90% for both devices for different FRRs, supporting the idea that the operating conditions strongly influence the performance of the devices. In addition, no correlation was found between FRR or TFR with the EE% values, which strongly suggests that the encapsulation process occurs randomly throughout the microfluidic device. However, the FRR is still an essential parameter in the size control of MLPs, which indicates that it is critical to define quality control strategies along with the nanoconjugates concentration [34]. Additionally, it is important to remark that the microfluidic synthesis of this type of drug delivery system provides a suitable route to enhance the therapeutic drug delivery efficiency compared with traditional methods. This provides further evidence for the relevance of the MLPs produced in this study, as they show consistent properties (e.g., size and morphology) without significant investments in infrastructure or instrumentation [34,50].

### 3.3. Magnetoliposomes Purification

**Figure 5:**
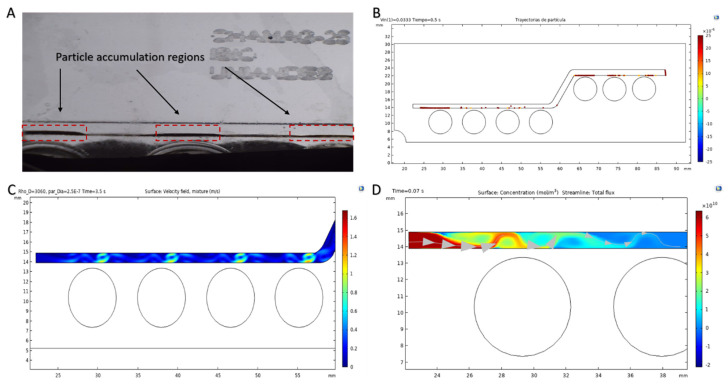
Qualitative results of the separation System 1. (**A**) Zoom of nanoconjugates accumulation regions inside the magnetophoretic microfluidic channel. (**B**) Particle trajectories in the magnetophoretic separation channel (nanoconjugates are shown in yellow and MLPs in red). (**C**) Velocity profile of the mixture. (**D**) Concentration profile in the microchannel.

Supplementary Figure S3A shows the intensity of the magnetic field acting on the microfluidic separation channel for System 1. The magnetic field is higher in proximity to the permanent magnets, as was expected [51]. Figure 5A shows the experimental performance of System 1 and the identification of some of the particles’ accumulation regions. Figure 5B shows the microfluidic system’s separation performance where both nanoconjugates and MLPs are attracted to the channel wall near the higher magnetic flux density regions. However, calculations failed to show that the percentage of nanoconjugates trapped is higher compared to the MLPs. Figure 5C presents the obtained velocity fields for the dispersed phase within the channels for the mixture model approach. The results indicate that an increase in the dispersed phase’s velocity matches each magnet’s highest intensity locations. Finally, Figure 5D shows the concentration profile for the nanoconjugates within the channel as estimated by the transport of diluted species model during the first seconds of the study. This result indicates that the concentration tends to increase in regions where the magnetic flux density is higher, which directly results in streamlines targeting these regions along the entire channel.

**Figure 6:**
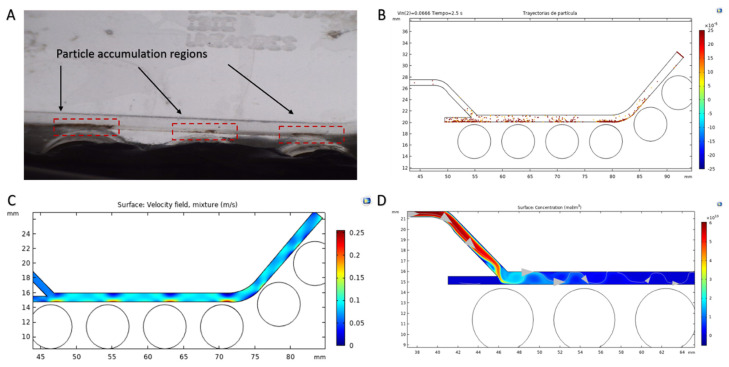
Qualitative results of the separation System 2. (**A**) Zoom of nanoconjugates accumulation regions inside the magnetophoretic microfluidic channel. (**B**) Particle trajectories in the magnetophoretic separation channel (nanoconjugates are shown in yellow and MLPs in red). (**C**) Velocity profile of the mixture. (**D**) Concentration profile inside the microchannel.

Supplementary Figure S3B shows the intensity of the magnetic field acting on the microfluidic separation channel for System 2. Figure 6A shows the experimental performance of System 2 and the identification of some of the particles’ accumulation regions. As for System 1, these regions are located in the channel sections near the magnets. Figure 6B shows the microfluidic system’s separation performance, where the behavior presented is almost identical to that of System 1. In the case of the results for the mixture model, Figure 6C illustrates the velocity field results for the dispersed phase within the channels. In contrast, Figure 6D shows that the concentration profiles for the nanoconjugates and their streamlines tend to increase in regions of high magnetic flux density.

Figure 5 and Figure 6 show qualitative results that illustrate the general performance of the proposed magnetophoretic separation devices and the trajectories of the nanoconjugates along the devices’ microchannels. Nevertheless, a quantitative analysis is critical to estimate separation efficiencies and further verify them experimentally. For the case of the mixture model analysis, we selected several locations (Supplementary Figure S4A,B) along the computational domain to determine the concentration of each type of particle component (i.e., MLPs and nanoconjugates) and to calculate their concentration difference close to the zones where the magnetic field is the highest. The final separation efficiency percentage (SE%) was calculated as the average percentage difference in concentration for all of the selected locations. For the particle tracing model, a particle counter for each type of particle was set at the system’s outlet.

**Figure 7:**
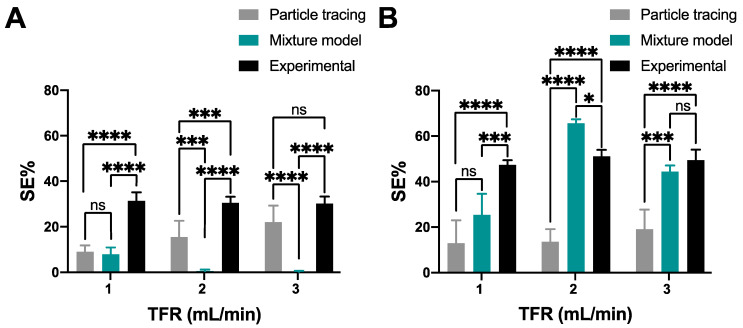
Microfluidic separation efficiency for Systems 1 and 2. (**A**) Comparison of both simulation approaches and the experimental results obtained for System 1 with a TFR ranging from 1 to 3 mL/min. (**B**) Comparison of both simulation approaches and the experimental results obtained for System 2 with a TFR ranging from 1 to 3 mL/min. Results with a *p*-value ≤ 0.05 (*) were considered significant. (*) corresponds to statistically significant difference with a *p*-value between 0.01 and 0.05; (***): 0.0001 ≤ *p*-value ≤ 0.001; (****): *p*-value < 0.0001; “ns” represents no statistically significant differences between the treatments.

The separation efficiency is calculated as the percentage difference between each type of particle arriving at the outlet. Figure 7 shows the quantitative results for the separation efficiency calculated for the two simulation approaches implemented here and the corresponding comparison with the obtained experimental results for both systems. The results show that the best separation efficiency achieved was 31.55% for System 1 at a TFR of 1 mL/min, while for System 2 it was 51.22% at a TFR of 2 mL/min.

For System 1, the results show the dominance of magnetophoretic over hydrodynamic forces, as an increase in the TFR led to a decrease in the separation efficiency [52]. Despite the higher separation efficiencies obtained with System 2, such a correlation was not clear for this system. In addition, it is important to highlight that we found that separation with small TFRs might lead to a relatively large fraction of MLPs trapped along with nanoconjugates in the accumulation regions, thereby reducing the number of purified MLPs at the end of the process.

Compared with the mixture model, quantitative results for System 1 indicate that the particle tracing model led to results with a higher level of agreement with those observed experimentally. In contrast, the System 2 mixture model simulation approach showed better performance in predicting quantitative separation results than those obtained with the particle tracing model. Despite these results, it is important to highlight a significant difference in the choice of one approach over the other in terms of ease of implementation. In this regard, although the mixture model describes the suspended magnetic nanoconjugates as a ferrofluid, making the simulation more realistic, the computational cost of this modeling approach compared with particle tracing is much higher [42,43,52]. Our results recommend implementing both models for a more comprehensive understanding of the devices, as these two approaches complement each other and might provide much more robust insights for further manufacturing and experimental testing.

Because the obtained separation efficiencies with System 2 were higher than System 1, it was selected to produce the MLPs for further experimentation.

### 3.4. In Vitro Testing of MLPs

#### 3.4.1. Biocompatibility

**Figure 8:**
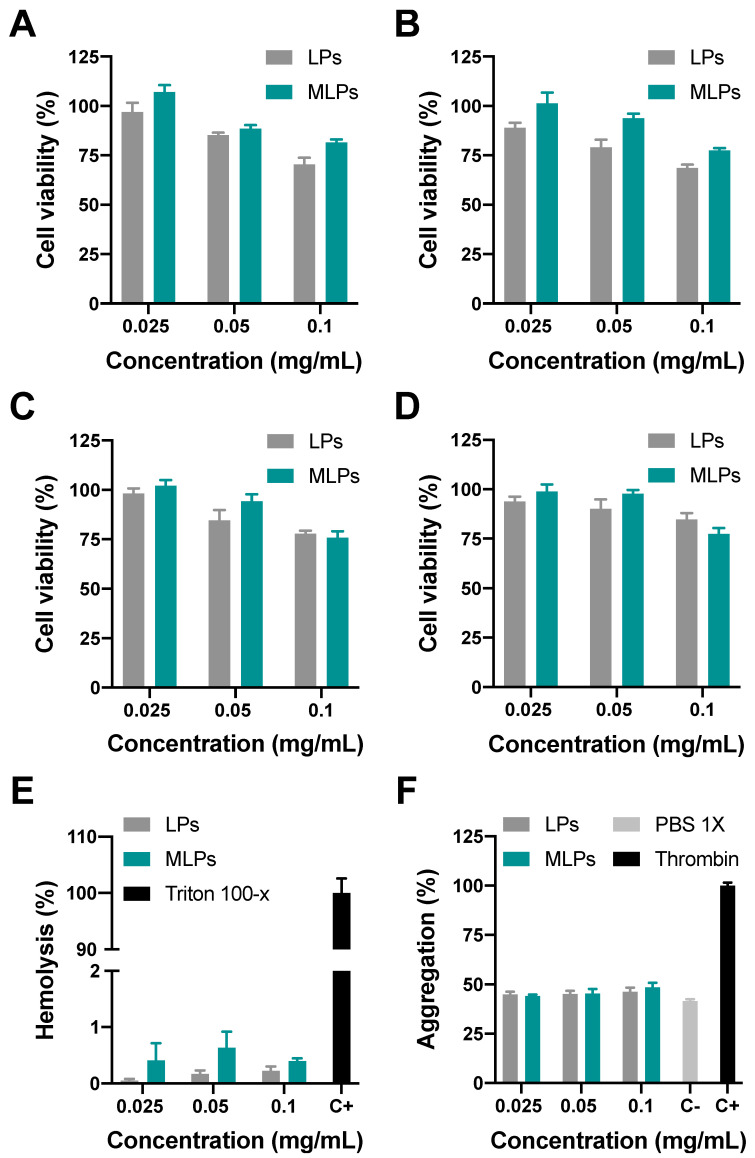
Biocompatibility assays for MLPs and LPs. Viability of Vero cells after 24 (**A**) and 48 h (**B**) of exposure. Viability of AGS cells after 24 (**C**) and 48 h (**D**) of exposure. (**E**) Hemolysis of MLPs and LPs with Triton X-100 as the positive control and PBS 1× as the negative control. (**F**) Platelet aggregation of MLPs and LPs with PBS 1× as the negative control and thrombin as the positive one.

Figure 8 shows the cytocompatibility, hemocompatibility, and platelet aggregation results for the produced MLPs and the LPs. Figure 8A,B shows the viability of Vero cells after 24 and 48 h of exposure to the different treatments. MLPs and LPs show high biocompatibility at concentrations below 0.1 mg/mL. However, at concentrations above 0.1 mg/mL, the cell viability decreases to about 70%, showing a dose-dependent behavior. A similar tendency was observed for AGS cells (Figure 8C,D). In addition, AGS cells exhibited high tolerance to the treatments, reaching viability percentages above 80%, even at concentrations higher than 0.1 mg/mL. Figure 5A–D shows the cytocompatibility results for Vero and AGS cells exposed to nanoconjugates. Significant cytotoxicity levels were found at concentrations above 50 µg/mL in AGS cells, whereas in Vero cells, the viability remained above 80% even at higher concentrations. This result can be related to the significant sensitivity of AGS cells to CTA. Additionally, Figure 8E and Supplementary Figure S5E show the hemolysis percentage of the MLPs, LPs, and nanoconjugates compared with the positive and negative controls. The results show hemolysis percentages below 1% for MLPs and LPs and 1.16% for the nanoconjugates at the highest evaluated concentrations. Similar results were obtained previously for MLPs, where the hemolysis percentages were below 5% [12,18,53,54]. Figure 8F and Supplementary Figure S5F show the platelet aggregation percentages of the MLPs, LPs, and nanoconjugates compared with the positive control. The results indicate that the platelet aggregation of MNP, MLPs, and LPs remain below 55% even at high concentrations. Compared with the negative control, the observed aggregation is acceptable even at the highest evaluated concentrations but is slightly higher than those reported previously [12,54].

#### 3.4.2. Cell Internalization and Endosomal Escape Analysis

**Figure 9:**
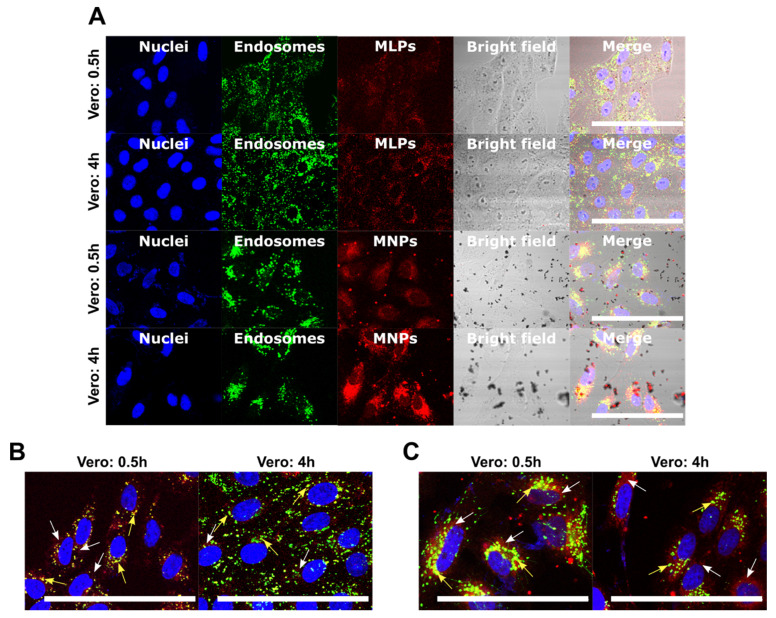
(**A**) Cell internalization and endosomal escape for magnetoliposomes (MLPs) and MNP-PEG-AEDP-CTA-RdB nanoconjugates (MNPs) in Vero cells with 40× magnification after 0.5 h and 4 h of exposure. The scale bar corresponds to 100 µm. Vero cells with 60× magnification and digital zoom to 120× after 0.5 h and 4 h of exposure to MLPs (**B**) and nanoconjugates (MNPs) (**C**). The scale bar for both (**B**,**C**) corresponds to 50 µm. The yellow arrows indicate colocalization between the green and the red channels, showing nanoparticles encapsulated into endosomes. The white arrows indicate non-colocalized zones, displaying nanoconjugates that escaped endosomes or reached the intracellular space by a different internalization mechanism.

**Figure 10:**
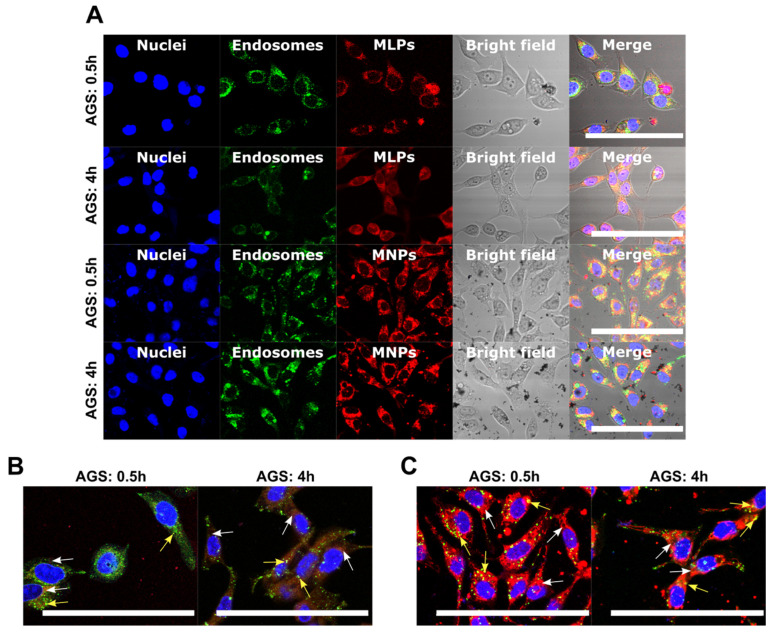
(**A**) Cell internalization and endosomal escape for magnetoliposomes and MNP-PEG-AEDP-CTA-RdB nanoconjugates (MNPs) in AGS cells with 40× magnification after 0.5 h and 4 h of exposure. The scale bar corresponds to 100 µm. AGS cells with 60× magnification and digital zoom to 120× after 0.5 h and 4 h of exposure to MLPs (**B**) and nanoconjugates (MNPs). (**C**). The scale bar for both (**B**,**C**) corresponds to 50 µm. The yellow arrows indicate colocalization between the green and the red channels, showing nanoparticles encapsulated into endosomes. The white arrows indicate non-colocalized zones, displaying nanoparticles that escaped endosomes or reached the intracellular space by a different internalization mechanism.

Figure 9 and Figure 10 show the confocal images corresponding to the delivery of MNP-PEG-AEDP-CTA-RdB nanoconjugates (labeled MNPs in the figures) and MLPs on Vero and AGS cells at different times (i.e., 0.5 h and 4 h). Staining with Hoechst 33,342 allowed determination of the impact of the treatments on the cell viability by analyzing the nucleus morphology and distribution. Images clearly show nuclei with regular spherical-shaped morphology with no visible fragmentation or DNA condensation [55]. These results demonstrate non-apoptotic cells, confirming high biocompatibility in both cell lines even after 4 h of exposure. These results provide further evidence of the high cell viability levels obtained via MTT (Figure 8A–D and Supplementary Figure S5A–D).

In addition, internalization and endosomal escape abilities were studied as a measure of the colocalization of Lysotracker Green^®^ with the rhodamine B labeled nanoconjugates and their distribution intracellularly. Figure 9 and Figure 10 show cells with a strong red fluorescent signal in the intracellular space, confirming the internalization of both MLPs and nanoconjugates. High cell penetration rates are most likely a consequence of employing PEG for the functionalization of MNPs to obtain the tested nanoconjugates and the use of LPs as powerful vehicles which favor membrane fusion and, consequently, the effective delivery of cargoes. LPs have also been reported to improve nanoparticle transport and plasma half-life [56]. The versatility of the developed vehicle allows the transport of CTA into the intracellular space even in the absence of LPs. This promising result presents the PEGylated magnetite-based nanovehicles as a fascinating tool for designing more potent oral delivery platforms to transport molecules of difficult intestinal absorption, such as CTA. This approach has been studied and validated previously by Kawish and colleagues [57].

**Figure 11:**
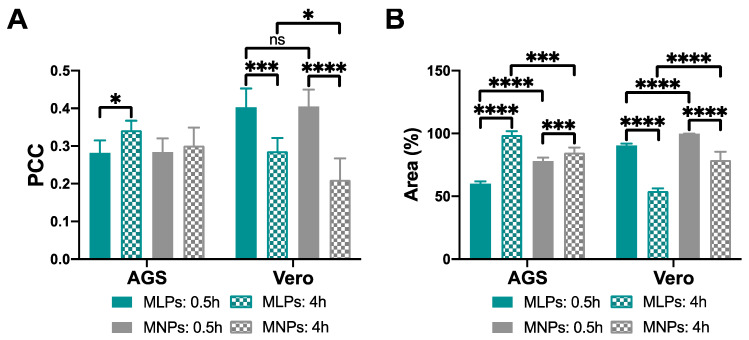
Pearson correlation coefficient (PCC) and percentage of area covered by the nanoconjugates in Vero and AGS cells. Higher PCC values indicate a higher amount of nanoconjugates trapped in endosomes. (**A**) PCC of MLPs and nanoconjugates in Vero and AGS cells for 0.5 h and 4 h. (**B**) Area covered by the MLPs and nanoconjugates in Vero and AGS cells for 0.5 h and 4 h. Results with a *p*-value ≤ 0.05 (*) were considered significant. (*) corresponds to statistically significant difference with a *p*-value between 0.01 and 0.05; (***): 0.0001 ≤ *p*-value ≤ 0.001; (****): *p*-value < 0.0001; “ns” represents no statistically significant differences between the treatments.

Figure 11 shows the quantitative results for the analysis of endosomal escape and the distribution of the nanoparticles into the cells. Pearson’s correlation coefficient (PCC) was used as a statistic tool for quantifying colocalization and covered area percentage to measure MLPs and nanoconjugates internalization and distribution. In AGS cells, a non-statistically significant difference was observed between the MLPs and the nanoconjugates, indicating that encapsulation into LPs failed to increase the endosomal escape of nanoconjugates. This was confirmed by an increase in the PCC after 4 h of exposure. However, the covered area of nanoconjugates slightly increased after 4 h, whereas the covered area of MLPs almost doubled for the same time. We hypothesize that these results might be a consequence of the interplay of different internalization routes. In this regard, as opposed to endocytic routes, it is very likely that nanoconjugates prevalently enter cells by a rapid and direct translocation mechanism [56]. In contrast, due to their negatively charged surface, LP and MLP internalization occurs mainly by endocytic routes. This is in line with recent reports that indicate that internalization rates for nanostructures with anionic coatings are lower than those of cationic and neutral coatings [56].

In Vero cells, the vehicles led to entirely different results, as evidenced by a statistically significant decrease in the PCC for both treatments after 4 h, confirming, therefore, endosomal escape. Somewhat surprisingly, for the same time, the covered area showed a statistically significant decrease. This suggests that, after internalization, the nanoconjugates escape endosomes and likely accumulate in different organelles. Future work will be dedicated to confirming this hypothesis.

The different penetration levels achieved for the two evaluated cell lines can be attributed to their significantly different cell membrane compositions, which might substantially alter the cell–nanoconjugate interactions. For example, the overexpression of claudin proteins in AGS cell membranes is likely to interfere with the internalization routes, rates, and achieved intracellular distributions [58]. Based on this, the rational design and development of novel vehicles for specific therapeutic applications must include a comprehensive analysis of membrane composition for the targeted cells. This is critical to engineer nanovehicles capable of taking advantage of and avoiding the possible interactions leading to cell penetration.

The obtained results clearly show the great potential of the developed nanovehicles as versatile carriers for difficult-to-transport drugs with high biocompatibility and the possibility to selectively internalize in cells with specific characteristics. Despite these promising results, future studies should include detailed studies on the impact of MLPs and the delivered compounds on microbiota bioactivity and bioavailability. This with the main objective of ensuring the homeostasis of patients’ microbiomes, which is controlled by molecular interactions and plays a central role in modulating metabolism, immunity, and response to infections. In addition, this understanding is critical to determining the bioavailability of the functional compounds, as it largely depends on the metabolism exerted by the microbiota during the delivery process [20,22]. We expect that our MLPs will show no impact on the microbiome balance and, therefore, contribute to novel oral delivery routes for patients suffering from complex diseases, such as eczema, inflammatory disorders, hypertension, and chronic kidney disease [20,59].

## 4. Conclusions

Over the past few years, MLPs have been studied to improve the cell penetration efficiency of nanovehicles for drug delivery after administration. This study presents a low-cost microfluidic device to produce MLPs with sizes ranging from 136.87 ± 3.97 nm to 787.47 ± 45.65 nm and polydispersity values ranging from 0.21 ± 0.02 to 0.58 ± 0.04. The device operates by putting into intimate contact lecithin liposomes (LPs) and functionalized magnetite nanoparticles (MNPs) within a serpentine microchannel. As reported elsewhere, the size of the MLPs appears to be strongly influenced by the flow rate ratio (FRR) between the components infused into the system, which supports the importance of considering this parameter for designing and optimizing the device’s performance. We prepared MNPs functionalized with a polymeric spacer (PEG) and a molecule containing a reducible disulfide bond (AEDP) to evaluate encapsulation. Additionally, we selected the immobilization of antibiotic CefTRIAxone (CTA) for proof-of-concept, considering its limited passing of the intestinal lumen after oral delivery. For all evaluated FRRs (i.e., from 1:1 to 5:1), the obtained nanoconjugates (i.e., MNP-PEG-AEDP-CTA) were encapsulated into the LPs (to form the MLPs) with efficiency above about 80% at a concentration of 0.17 mg/mL and while operating the device at a TFR of 5 mL/min.

However, this approach is challenging, as purifying the obtained MLPs is not a simple task, due to the proximity in properties of the involved components. Here, we decided to take advantage of the magnetic properties of the nanoconjugates to develop a robust and high-throughput separation scheme enabled by microfluidics and permanent magnets. Accordingly, we designed two magnetophoretic microfluidic separation devices where permanent magnets can be located along the main separation channels to retain excess nanoconjugates. To investigate the feasibility of this approach, we conducted two different multiphysics simulations approaches that provided complementary qualitative and quantitative information regarding the purification efficiency of the proposed devices. The first one was based on a particle tracing model, while the second relied on a multiphase mixture model. After conducting parametric sweeps for the FRRs, the results of the two approaches allowed us to confirm that the designed systems were well-suited to retain the nanoconjugates at hot spots of high magnetic field intensity. Although the qualitative results of both simulations show adequate behavior for the nanoconjugates within the systems, quantitative results varied considerably between approaches, due to the differences in inlet parameters and conditions used for each approximation. Conversely, it is highly suggested to combine these approaches for the rational design of microfluidic separation devices. The experimental testing showed separation efficiencies ranging from 30% to 31% for System 1 and 47% to 51% for System 2, showing a weak correlation between the TFR and separation efficiency in System 1. According to these results, System 2 operating at a TFR of 2 mL/min was employed for purifying MLPs for further in vitro testing.

Finally, we validated and demonstrated the great potential of the developed nanovehicles as a versatile carrier for difficult-to-transport drugs by showing high hemocompatibility, low platelet aggregation, and high cytocompatibility in two relevant cell lines (i.e., Vero and AGS). Despite different cell internalization and endosomal escape results in these two cell lines, the achieved coverage shows a promising potency, which is attractive for applications in gastrointestinal delivery. Furthermore, the results suggest unique nanoconjugate–cell membrane interactions and, consequently, interplay of different internalization mechanisms, which need to be considered for further surface engineering experiments.

## Figures and Tables

**Table 1:** Parameters employed for the particle tracing module.

Parameter	Value	Units
$B_{r}$	1.00	T
μ	1.00 × 10^−3^	Pa.s
$\mu_{0}$	12.57 × 10^−7^	H/m
$\mu_{\mathrm{rMNps}}$	2.50	DV
$\mu_{\mathrm{rMLs}}$	1.50	DV
$\rho_{\mathrm{MNps}}$	5180	kg/m^3^
$\rho_{\mathrm{MLs}}$	3063	kg/m^3^
$d_{\mathrm{MNps}}$	1.00 × 10^−7^	m
$d_{\mathrm{MLs}}$	2.50 × 10^−7^	m

**Table 2:** Parameters employed for the mixture model.

Parameter	Value	Units
$B_{r}$	1.00	T
μ	1.00 × 10^−3^	Pa.s
$\mu_{\mathrm{rMNps}}$	2.50	DV
$\mu_{\mathrm{rMLs}}$	1.50	DV
$\mu_{0}$	12.57 × 10^−7^	H/m
$\rho_{C}$	1000	kg/m^3^
$\rho_{\mathrm{MNps}}$	5180	kg/m^3^
$\rho_{\mathrm{MLs}}$	3063	kg/m^3^
$d_{\mathrm{MNps}}$	1.00 × 10^−7^	m
$d_{\mathrm{MLs}}$	2.50 × 10^−7^	m
$D_{\mathrm{MNps}}$	4.83 × 10^−12^	m^2^/s
$D_{\mathrm{MLs}}$	1.93 × 10^−12^	m^2^/s
$\phi_{d}$	0.20	DV
$x_{\mathrm{MNPs}}$	4.00 × 10^−4^	m^3^/kg
$x_{\mathrm{MLs}}$	1.33 × 10^−4^	m^3^/kg

## Supplementary Materials

The following supporting information can be downloaded at: https://www.mdpi.com/article/10.3390/pharmaceutics14020315/s1, Figure S1: Manufactured separation Systems 1 and 2. (A) System 1 (B) System 2, Figure S2: Meshing used for multiphysics simulations. (A) System 1 for particle tracing; (B) System 2 for particle tracing; (C) System 1 for mixture model; (D) System 2 for mixture model, Figure S3: Magnetic flux density results for the separation Systems 1 and 2. (A) System 1 and (B) System 2, Figure S4: Evaluation points for mixture model simulations separation efficiency. (A) System 1 and (B) System 2, Figure S5: Biocompatibility assays for nanoconjugates (MNPs). Viability of Vero cells after 24 (A) and 48 h (B) of exposure. Viability of AGS cells after 24 (C) and 48 h (D) of exposure. (E) Hemolysis of MNPs with Triton X-100 as the positive control and PBS 1× as the negative control. (F) Platelet aggregation of nanoconjugates with PBS 1× as the negative control and thrombin as the positive one.
