# Supplementary material for: Microfluidic Synthesis and Purification of Magnetoliposomes for Potential Applications in the Gastrointestinal Delivery of Difficult-to-Transport Drugs
Source: Pharmaceutics. 2022 Jan 28;14(2):315. doi: 10.3390/pharmaceutics14020315 (PMC8877506; doi:10.3390/pharmaceutics14020315)
Supplement: Supplementary file 1 [file pharmaceutics-14-00315-s001.zip › pharmaceutics-1507273-supplementary.pdf]

pharmaceutics

MDPI

Supplementary Materials: Microfluidic Synthesis and Purification of Magnetoliposomes for Potential Applications in the Gastrointestinal Delivery of Difficult-to-Transport Drugs

Carlos E. Torres, Javier Cifuentes, Saúl C. Gómez, Valentina Quezada, Kevin A. Giraldo, Paola Ruiz Puentes, Laura Rueda-Gensini, Julian A. Serna, Carolina Muñoz-Camargo, Luis H. Reyes, Johann F. Osma and Juan C. Cruz

Supplementary information

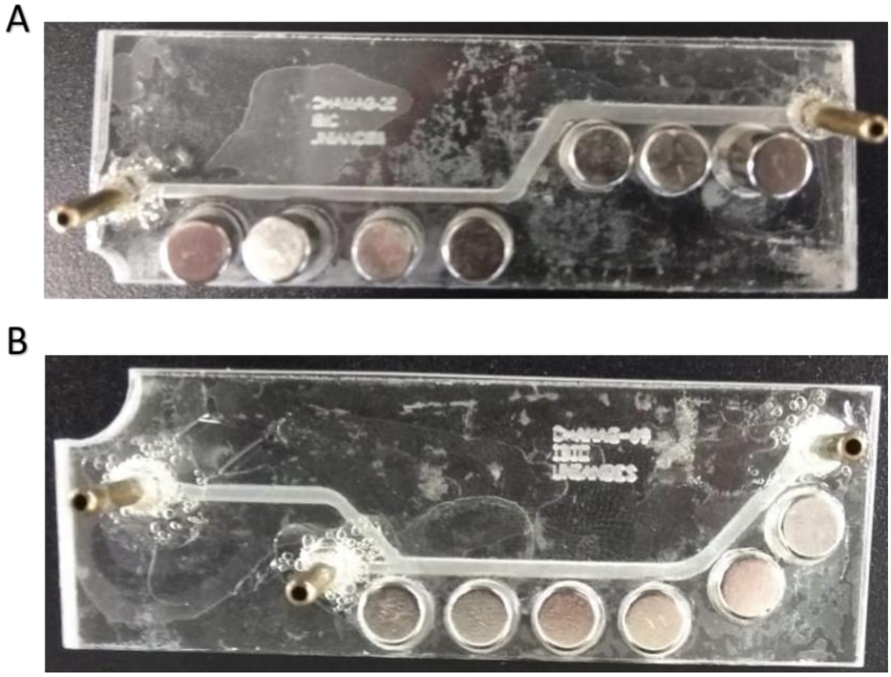

Figure S1. Manufactured separation Systems 1 and 2. (**A**) System 1 (**B**) System 2.

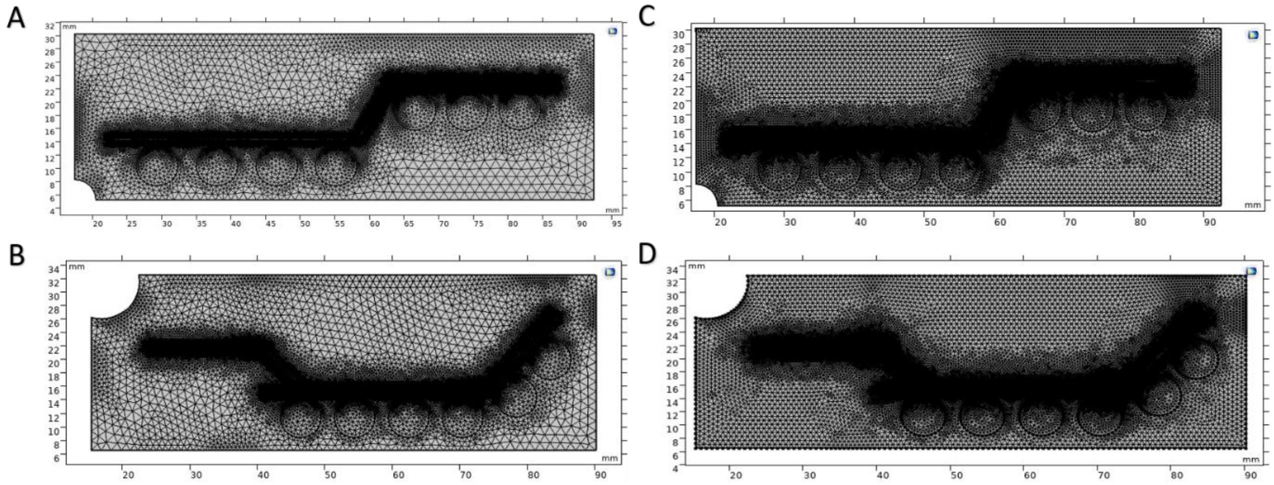

Figure S2. Meshing used for multiphysics simulations. (**A**) System 1 for particle tracing; (**B**) System 2 for particle tracing; (**C**) System 1 for mixture model; (**D**) System 2 for mixture model.

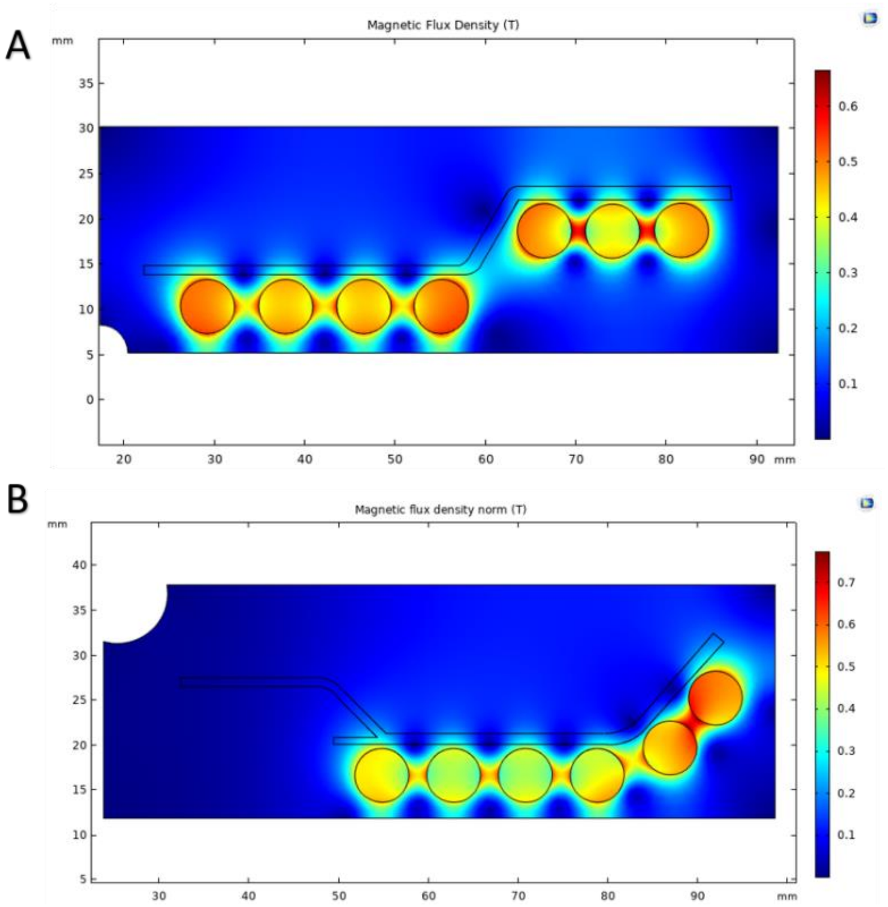

Figure S3. Magnetic flux density results for the separation Systems 1 and 2. (**A**) System 1 and (**B**) System 2.

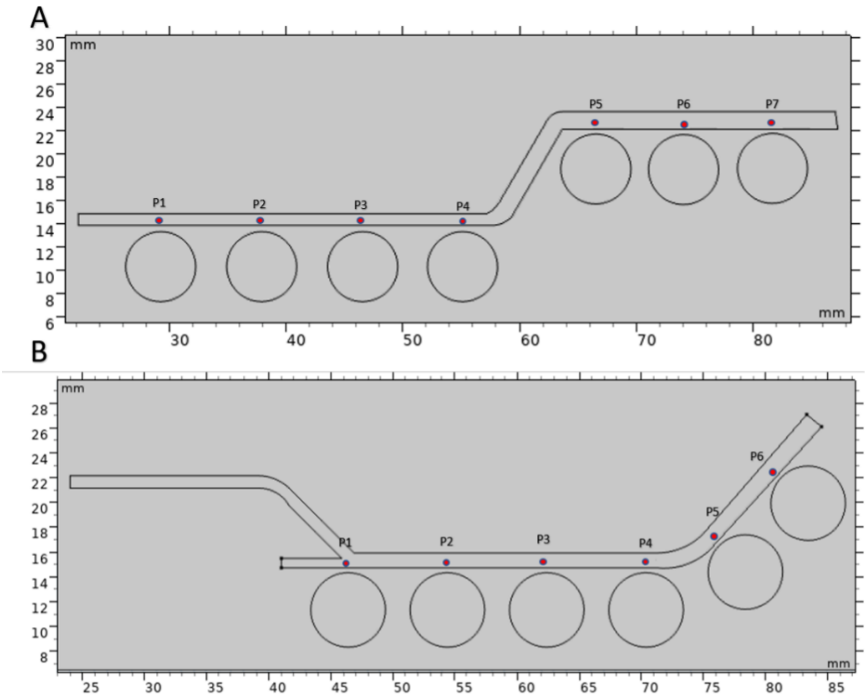

Figure S4. Evaluation points for mixture model simulations separation efficiency. (**A**) System 1 and (**B**) System 2.

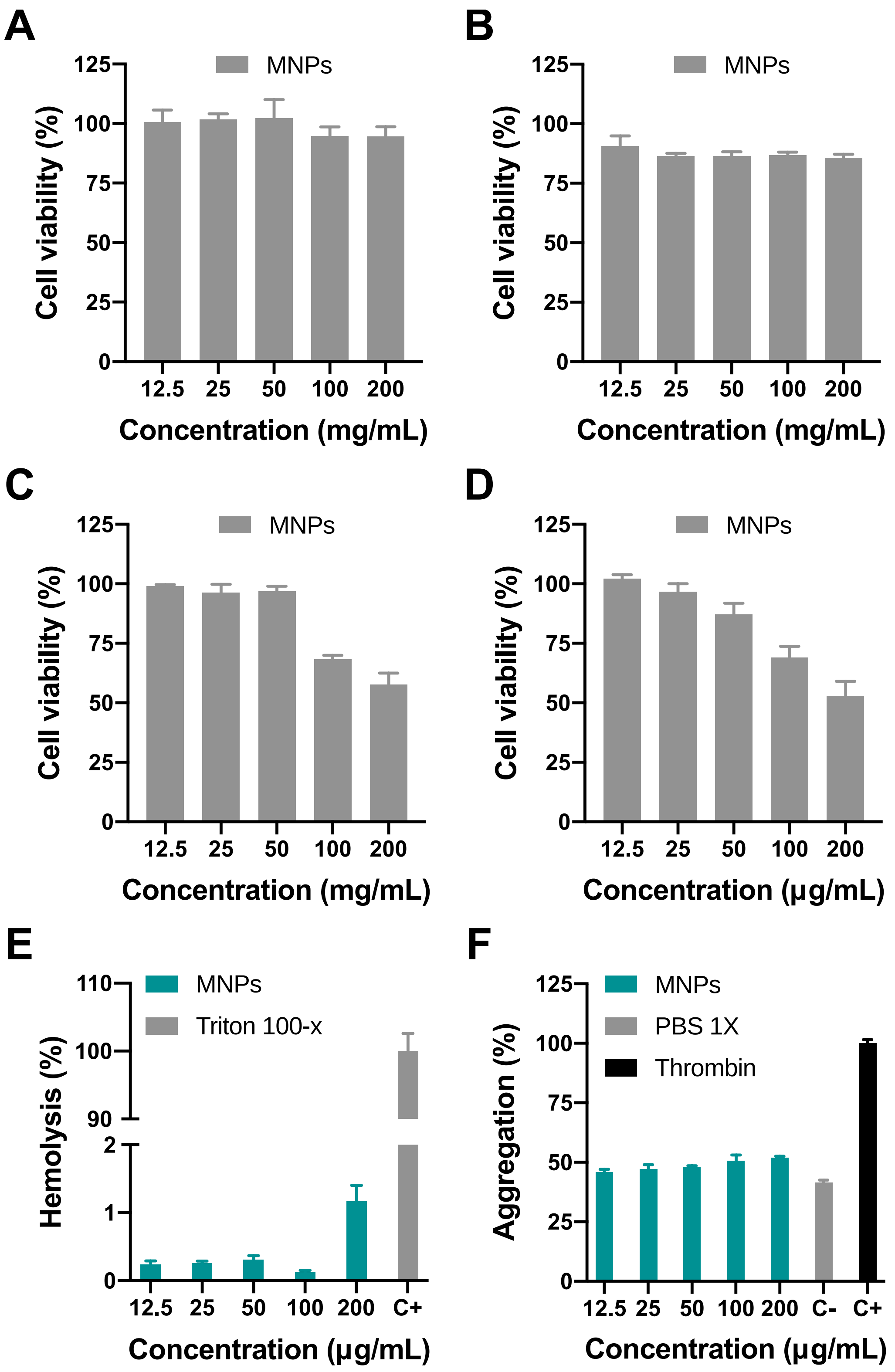

Figure S5. Biocompatibility assays for nanoconjugates (MNPs). Viability of Vero cells after 24 (**A**) and 48 h (**B**) of exposure. Viability of AGS cells after 24 (**C**) and 48 h (**D**) of exposure. (**E**) Hemolysis of MNPs with Triton X-100 as the positive control and PBS 1× as the negative control. (**F**) Platelet aggregation of nanoconjugates with PBS 1× as the negative control and thrombin as the positive one.
